# Patient experiences in psychiatric departments for the elderly (PEPDE): development, properties, and use of a brief questionnaire

**DOI:** 10.1186/s12888-023-04633-y

**Published:** 2023-03-16

**Authors:** Torleif Ruud, Ingrid Kyte Fjellestad, Ketil Hanssen-Bauer

**Affiliations:** 1 Division Mental Health Services, Akershus University Hospital, Box 1000, Lørenskog, 1478 Norway; 2 Institute of Clinical Medicine, Faculty of Medicine, University of Oslo, Oslo, Norway

**Keywords:** Patient experiences, Inpatient department, Elderly, Psychogeriatric, Questionnaire

## Abstract

**Background:**

Measuring patient experiences at psychiatric inpatient departments for the elderly need measurements adapted to the situation and challenges of the age group. We did not find any such instrument. The aim of this study then was to develop and document the measurement properties of a reliable and valid questionnaire about experiences of patients without severe neurocognitive disturbances at psychiatric inpatient departments for the elderly, which can be used in quality improvement and research.

**Methods:**

Aiming for good content validity, we developed a questionnaire with 37 questions based on a review of the literature on important aspects for the elderly in psychiatric inpatient departments and on feedback from elderly patients from other questionnaires developed for use in psychiatric departments for adults. Using this first questionnaire, we collected data from 151 patients in psychiatric departments for the elderly in nine health trusts. We then revised the questionnaire based on comments from patients and interviewers on the questions, and we decided which questions we would keep and which we needed to adjust to improve clarity. This resulted in a final questionnaire of 20 questions. We analyzed the internal structure (factors and their internal consistency) of this final questionnaire based on data collected from a new sample of 96 patients. To test the construct validity of the questionnaire, a sample of 26 service user representatives, clinicians and researchers were asked to sort the questions based on identified factors.

**Results:**

The final questionnaire consisted of 20 questions giving a valid and reliable measurement tool with four subscales: Patient-centered Interaction, Outcome, Care and Safety, and Information on Rights. Very few unanswered questions indicate that the questionnaire is feasible, as patients seemed to understand the questions and the response scales well. It is desirable that structural validity is confirmed with a larger sample.

**Conclusion:**

Our final questionnaire “Patient Experiences in Psychiatric Departments for the Elderly” (PEPDE) has adequate measurement properties and seems to be well understood. It can be used as a questionnaire or an interview for quality improvement and research.

**Supplementary Information:**

The online version contains supplementary material available at 10.1186/s12888-023-04633-y.

## Background

Patient health care experiences have increasingly been included as an important aspect in assessment and improvement efforts of health services in the last two decades. Patient-reported experience measures (PREMs) are tools that capture “what” happened during an episode of care and “how” or “how often” it happened from the patient’s perspective [1–3]. The terms patient satisfaction and patient experience are distinct but related concepts. However, they are often used interchangeably, and the nature and direction of their relationship continues to be debated [1].

Recent systematic reviews summarized the information on PREMs that are now available. A systematic review of the validity and reliability of 88 PREMs developed in all areas of medicine since 1993 found ten PREMs for mental health services [3]. Four of these were for inpatient services, but none were for services for the elderly. A systematic review of PREMs specifically in mental health care for adults identified 75 PREMs [4]. Seven key domains were found across the measures: interpersonal relationships, respect and dignity, access and care coordination, drug therapy, information, psychological care, and care environment. However, one of the exclusion criteria was measures designed especially for the elderly. Two reviews of measurement tools for patient satisfaction in mental health services identified similar domains [5, 6]. However, none of the identified measurement tools in these two reviews were for psychiatric inpatient departments for the elderly. Thus, the reviews did not identify any PREMs regarding such psychiatric inpatient departments for the elderly. Reasons for this may be that there are few, if any, such measurement tools and that most reviews excluded measurement tools for the elderly [4]. While it may be possible that instruments for adults to some extent may be suitable also for elderly patients, this seems so far not to have been studied or proved.

We found very few studies on patient experiences in psychiatric inpatient departments for the elderly, none of them done recently. A study in a psychiatric department for the elderly in a psychiatric hospital was done as qualitative interviews focusing on seven critical aspects of how patients in a general hospital ward experienced their treatment [7]. Most of the 69 participating patients had felt respect from the staff, found the staff coordinated, received useful information, been well taken care of, felt emotional support and that their family had been involved, were prepared for discharge, and benefited from the stay.

In another study conducted in a psychiatric department for the elderly, a questionnaire on patient satisfaction developed for adult inpatients was used [8]. These elderly patients reported feeling the least satisfied with the information about different treatment options. Just over half of the patients thought the information about mental illness and diagnosis was “good” or “very good,” whereas one in six answered it was “bad.” There was also dissatisfaction with the information on appeal options and about the effects and side effects of medications. The study concluded that important aspects to investigate further included patients’ access to information, understanding of treatment, participation opportunities, perceived benefit of the ward environment, and experience of the discharge process.

Conditions important and relevant for elderly inpatients may be different than those for working age adult inpatients [9]. Questionnaires or interviews on elderly inpatients’ experiences need to build upon what we know is important for these patients. The relationship between patients and staff was considered the most important therapeutic factor during stays in psychiatric inpatient departments for adults [10, 11]. This was also found in a study from a psychiatric inpatient department for the elderly [7].

Knowledge of what aspects are the most important for inpatients in psychiatric services is the key to asking the right questions regarding their experiences as inpatients. This was done in a German study where 80 adult inpatients in psychiatric departments answered questions about the importance of and satisfaction with 92 different aspects of the services provided [12]. Yet inpatients in wards for the elderly were excluded, and we have not found any similar study about what aspects are both important and satisfying for elderly inpatients. In a qualitative Norwegian study, importance was the only angle explored. Nine inpatients from four psychiatric departments for the elderly described what was important for them during their stay [13]. These aspects were relations to the staff and other patients, a holistic treatment (activities for patients, the social milieu at the ward, planning the discharge), and information and influence (knowledge about their illness, participation in treatment decisions).

Measuring elderly patients’ experiences of psychiatric inpatient stays can be difficult because several factors can affect how the elderly respond and whether they can answer the questions. Patients may have mental problems, such as anxiety, depression, cognitive impairment, psychosis, and confusion. They may also have a somatic disease in which various functions are affected by aging. Questionnaires surveying the experiences of mental health inpatient care most often do not include the psychiatric wards for the elderly [14, 15]. Possible causes for this may be challenges regarding participant selection, difficulties with filling in self-completion questionnaires for some patients, or other issues regarding data collection methods.

We have found no established PREM for measuring patient experiences in psychiatric inpatient departments for the elderly, and there are few studies and little information on the experiences of this group of patients. Developing such a PREM to start filling this gap must therefore build on the present knowledge of adult patient experiences in psychiatric inpatient departments and still aim to address issues that may be important for the elderly and give the measurement tool a form suitable for the elderly.

## Methods

### Aim of the study

The aim of this study was to develop and document the measurement properties of a reliable and valid questionnaire measuring experiences of patients without severe neurocognitive disturbances at psychiatric inpatient departments for the elderly and to make it available for use in quality improvement and research.

### Context

This study was done at the Department for Research and Development in the Division of Mental Health Services of Akershus University Hospital, Lørenskog, Norway, in collaboration with the Psychiatric Department for Elderly and similar departments contributing to data collection in other health trusts throughout the country.

The project started with a local pilot study in 2007 on inpatients’ experiences in the Psychiatric Department for the Elderly. In this pilot, we tested a questionnaire developed for national surveys of patient experiences in psychiatric wards for adults [16, 17] and an interview for the same patient group recommended by the national health authorities [18]. However, we found that both these measurement tools had questions and concepts that did not suite elderly patients’ situations and that use of several different response scales in the same instruments placed too much demand on the respondent’s adaptability. We searched but did not find any questionnaire or structured interview designed to measure elderly patient experiences in psychiatric inpatient departments, so we decided to develop such a questionnaire.

### Design

**Fig. 1:**
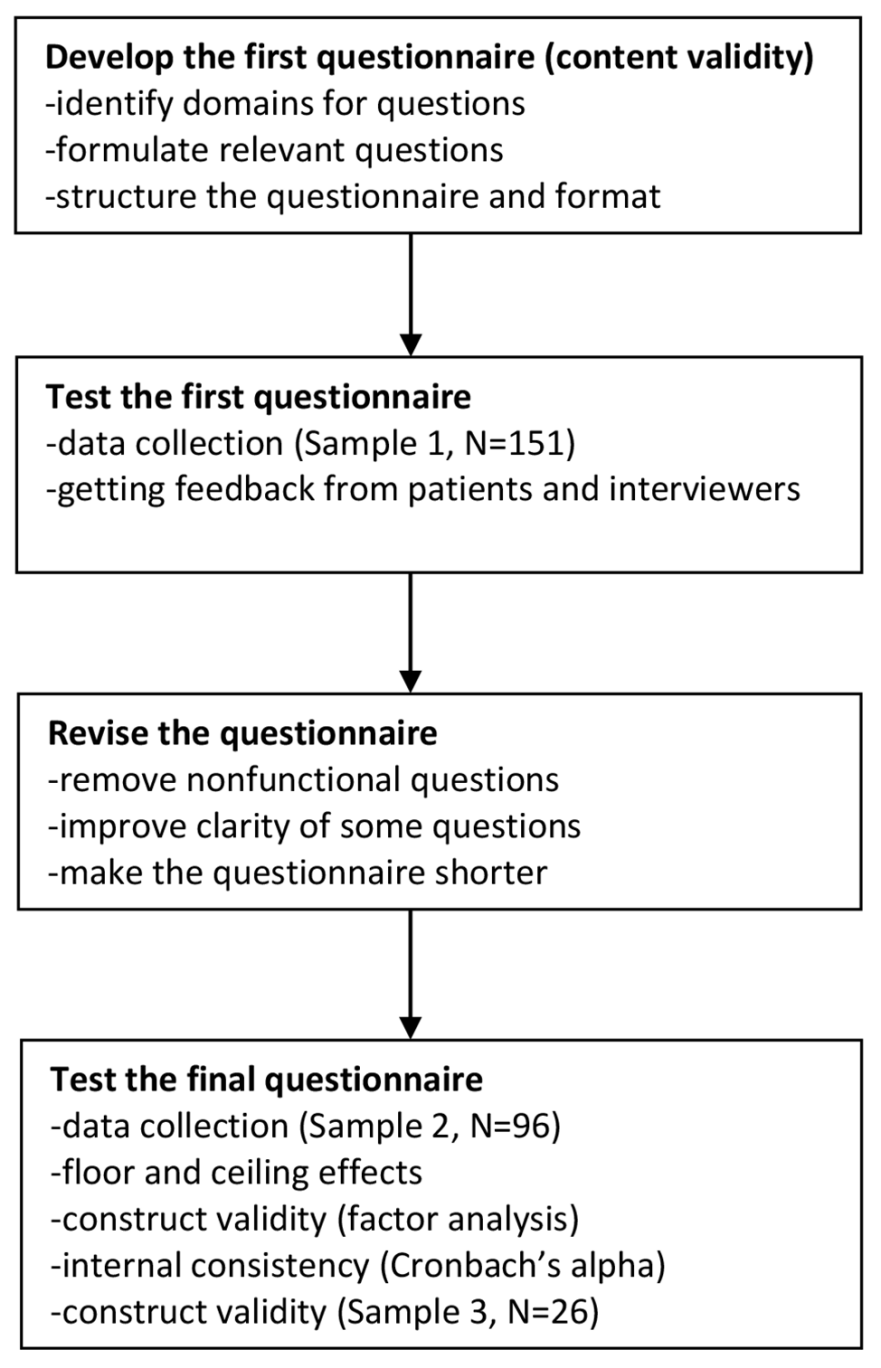
Stages in development and testing of the questionnaire Patient Experiences in Psychiatric Departments for the Elderly (PEPDE)

We did the development and testing in four stages, as shown in Fig. 1. The stages were developing the first questionnaire, testing the first questionnaire, revising the questionnaire, and testing the measurement properties of the final questionnaire. All this was done as recommended in the literature on the development and testing of measurement tools [19, 20].

Based on the COSMIN standards for systematic reviews of patient-reported outcome measures, we reported on the content validity of questions, structure and internal consistency of the factors in the questionnaire, and construct validity of subscales based on these factors [21–24]. The COSMIN standards were developed for patient-reported outcome measures (PROMs). However, as there are no such standards yet for PREMs, COSMIN has also been used in systematic reviews of PREMs [3, 6].

### Development of the first questionnaire

We developed a list of aspects and a group of questions for each, building on patient feedback on the instruments used in the pilot study, clinicians’ experiences, literature on aspects important to the patient group, and our knowledge and experience using the two instruments in the pilot study. The clinicians in the project and an advisory group of clinicians and health workers from several psychiatric departments for elderly in the eastern part of Norway collaborated, formulating and discussing questions thoroughly to make them relevant and understandable to the patients. We aimed to find relevant and clear questions and organized them into topics with a logical sequence for an inpatient stay. All but three questions had a five-point response scale (1 = ’not at all’, 2 = ‘to a small extent’, 3 = ‘to some extent’, 4 = ‘to a large extent’, 5 = ’to a very large extent’) with an additional response of “not relevant” for some questions. Patients had the response scale visualized on a separate sheet during the interview. Three questions had a dichotomous response (yes/no).

### Samples and data collection

Sample 1 for testing the first questionnaire was data from a cross-sectional multicenter study collecting data from 2009 to 2011 in psychiatric inpatient departments for the elderly in 9 of the 19 health trusts in Norway. We invited male and female patients aged 65 and over who were ready for discharge after a stay of seven days or more to participate. They had to be able to communicate verbally, understand the interview questions, remember well enough to give rational answers, and give written informed consent to participate. Exclusion criteria were the inability to give consent and significant cognitive impairment scoring below 25 on the screening tool Mini Mental State Evaluation, Norwegian version (MMSE-NR), which is a test of cognitive functions [25, 26]. A total of 151 patients (median 17 patients per department, range 10–23) participated. Mean age was 75 (SD 7) years. Thirty (20%) were 60–69 years old, 83 (55%) were 70–79 years old, 35 (23%) were 80–89 years old, and 3 (2%) were 90–99 years old. The sample consisted of 108 (72%) women and 43 (28%) men. Eighty (63%) of the patients were living alone. Median length of stay was 7 weeks (range 1–29 weeks), and 16 patients (11%) had an involuntary admission. We considered the participants to be representative of patients in psychiatric inpatient departments for the elderly in Norway.

Sample 2 for testing the factor structure of the final questionnaire was data from 96 questionnaires completed from 2015 to 2018 in the Psychiatric Department for the Elderly at Akershus University Hospital to collect feedback from patients and use this in the further development of the department. After approval from the Regional Committee for Medical and Health Research Ethics for the South-Eastern Health Region and the privacy ombudsman for our hospital, we received anonymous data from 96 elderly inpatients meeting our original inclusion criteria described above for Sample 1. Characteristics of sample 2 could not reported as these variables were not in included in the anonymous data we received from the ward.

Sample 3 for testing construct validity of the final questionnaire in 2020 consisted of 26 people. Four were members of the Service User Council in our university hospital, thirteen were clinicians or health workers in the psychiatric departments for the elderly in our university hospital or in a health trust in Northern Norway, and nine were researchers in the mental health research department in our university hospital or The Norwegian National Centre for Ageing and Health. All were experienced health professionals or researchers and were recruited by their leaders on request by email from our project group. The participants had no previous knowledge of the questionnaire. Using an online form, they completed sorting the questions based on the factors identified in the factor analysis.

### Data analyses

We used descriptive statistics to inspect the distribution of responses from Sample 2 on all questions in the final questionnaire. We imputed missing responses with the mean value of each question and conducted an exploratory factor analysis of the questions with a graded response scale to examine the factor structure of the final questionnaire [19, 20, 27]. We used principal components analysis with varimax rotation and Kaiser’s criteria with eigenvalue of more than 1, including questions with loadings of 0.40 or more in the factor with the highest loading of the question. We then calculated Cronbach’s alpha for each factor and interpreted the degree of internal consistency based on suggested guidelines as unacceptable (alpha below 0.70), fair (alpha 0.70 to 0.79), good (alpha 0.80 to 0.89), and excellent (alpha 0.90 and above) [28]. The corrected item-total correlation was analyzed for each item and was expected to be above 0.50 for all items [20].

To determine construct validity of the final questionnaire, we asked participants (Sample 3) without any previous knowledge of the questionnaire to sort the questions on the identified factors using an online form. Participants were then to sort each question to the factor they felt it belonged to [19, 20]. Our hypothesis was that their sorting of the questions would be similar to the sorting made by the factor analysis, examining responses by comparing the distribution (percentage) of each question based on the factors.

We analyzed floor and ceiling effects with 50% as the cut-off criterion, considering a question acceptable if fewer than 50% of the patients had chosen the most negative or the most positive answer, respectively [29, 30]. All data analyses were done with SPSS for Windows (version 22 in 2013 and version 26 in 2020).

### Testing the first questionnaire

The questionnaire was tested with data collected from Sample 1. A day or two before discharge, health personnel with special training conducted interviews in a standardized manner, reading out the questions and scoring responses as they appeared in the questionnaire. Patients were given copies of the questionnaire and response scale and encouraged to follow along with the reading. When scoring, the interviewer aimed to detect and resolve any ambiguities or misunderstandings in patient responses. Both the patient and the interviewer were also asked for comments on how each question functioned in the interview, and these were recorded in an appendix to the questionnaire. An interview took 30 to 45 min, with some additional time for writing the patient’s and interviewer’s comments. Scores from the questionnaire were entered into an electronic database with a code number representing each patient. The code key was securely stored in the psychiatric department for the elderly where the patient was, and the code key was deleted after the completion of the data collection.

### Revising the first questionnaire

We did a systematic revision of the first questionnaire in 2013 with the steps described below, based on recommended methods for questionnaire development [19, 20]. First, we removed questions that did not work satisfactorily according to comments given by patients and interviewers. Second, we reviewed the remaining questions for their importance of content based on what topics patients in psychiatric inpatient departments for the elderly reported as important for the quality of their stay [13]. Then we decided which of the remaining questions were most important to retain. Because many patients had commented that the first questionnaire was far too long, questions covered better by other related questions were removed, changing the new and final questionnaire to 20 questions that would fit on one page and be easier to complete. Third, we improved the clarity of long questions by removing redundant words, making the question shorter and easier to understand without changing its meaning. The final 20 questions also included questions we considered essential and easy to understand without any changes.

### Testing content validity

We did an assessment of content validity using the ten COSMIN criteria for content validity [24]. These criteria are grouped under three main concepts: Relevance (five criteria), Comprehensiveness (one criterion), and Comprehensibility (four criteria). Developing the questionnaire and getting detailed comments from patients and interviewers in testing the first questionnaire, we also had much focus on achieving an adequate content validity, as described above and below.

### Testing structural validity, internal consistency, and construct validity of the final questionnaire

The final questionnaire has been used routinely since 2014 in the Psychiatric Department for the Elderly at Akershus University Hospital to collect feedback from patients for further development of the department. To analyze the factor structure (structural validity) and internal consistency of the factors in the final questionnaire, we used anonymized data from Sample 2 as described under Data analyses. Furthermore, construct validity was tested with data from Sample 3 as described under Data analyses.

## Results

### Content of the questionnaire

The first questionnaire consisted of 37 closed questions organized into the following seven sections: general experiences, outcomes, how they experienced the staff, information, information on patient rights, cooperation with relatives, and discharge preparation and importance of admission. The questionnaire was tested using data from Sample 1, as described above, including feedback from patients and interviewers. An English translation of the 37 questions is shown in online Additional Table A.

The patients confirmed that the content of most questions was relevant and important. They and the interviewers gave valuable information on which questions were difficult to understand or less relevant. They also gave clear feedback that the questionnaire was too long. We also discussed the questionnaire and the preliminary results with representatives from participating departments who gave similar comments. We first removed nine questions that several patients found difficult to answer or not important. These included three questions about what information family/relatives had received as several patients did not know this, did not want relatives to be informed, or had no close relatives. To reduce the questionnaire to 20 questions, we also removed eight questions that were less important or better covered by other questions. Of the remaining 20 questions, we shortened and improved the clarity of six questions and kept 14 without making any changes. Online Additional Table A outlines revision results and decisions for each of the 37 questions from the first questionnaire.

Table 1 shows the distribution of responses for the 20 questions from the 96 patients in Sample 2 who completed the final questionnaire. The data from Sample 2 using the final questionnaire had a total of 32 (1.7%) missing responses spread over 17 questions. The number of missing responses for each question is shown in Table 1. The distribution of missing responses on the 20 questions seems to be random, with no missing on three questions, one missing on eight, two missing on five, three missing on three, and five missing on one. The scarcity of missing responses shows that the questions and response scale were well understood by patients and that the questionnaire may be considered feasible. None of the questions had a positive floor effect, where 50% or more of the patients had chosen the lowest response (“not at all”), and none had a positive ceiling effect, where 50% or more of the patients had chosen the highest response (“to a very large extent”).

**Table 1:** Frequency distribution of responses for the final questionnaire (N = 96)

Questions with a graded response scale	Not at all	To a small degree	To some degree	To a large degree	To a very large degree	Missing
1.Were you involved in planning your stay?	38	16	18	15	8	1
2.Have you been consulted about the treatment?	20	16	30	22	8	0
3.Has your doctor or psychologist been able to help you?	3	9	28	33	22	1
4.Have nurses and other health personnel been able to help you?	2	3	25	46	19	1
5.Has your mental health improved?	13	11	24	32	13	3
6.Has your physical health improved?	20	13	32	20	10	1
7.Have you become better able to master your daily tasks?	13	9	28	31	12	3
8.Have you felt safe in the department?	2	0	10	51	31	2
9.Have you received information about how the therapists assessed your health condition?	10	18	25	32	8	3
10.Have you received information about treatment options available to you?	15	20	26	19	14	2
11.Have you received information about the effects of the medication?	14	23	30	20	8	1
12.Have you received information about possible medication side effects?	31	22	25	11	5	2
13.Has the information been understandable?	6	13	24	35	13	5
14.Have the health personnel treated you with respect?	0	1	4	59	31	1
15.Have you been involved in preparing your discharge?	12	14	27	28	13	2
16.Do you feel ready to be discharged now?	9	11	21	33	20	2
17.Would you return here if you needed a new stay?	4	3	14	36	38	1
**Questions with a dichotomous response scale**				**No**	**Yes**	**Missing**
18.Have you received information about your right to access your medical record?				69	27	0
19.Have you been informed that you have opportunity to complain about the treatment?				66	30	0
20.Have you received information about your right to an individual plan?				72	23	1

### Content validity

Our assessment of content validity was done for three aspects using the ten COSMIN criteria for this [24]. We found the questionnaire relevant (for the construct of interest, for the target population, for the context, with an appropriate response scale, and with an appropriate recall period), fairly comprehensive (including most key concepts), and comprehensible for the patients (understandable instructions, understandable questions and response scale, appropriately worded questions, and with a response scale matching the questions). We considered the content validity to be adequate from our assessment.

### Structural validity and internal consistency

The final questionnaire had adequate structural validity with three factors from the 17 questions with graded response scales, as shown in Table 2. The internal consistency was good (Cronbach’s alpha of 0.80 or above) for two factors and fair (alpha of 0.70 or above) for one factor. The corrected item-total correlation reported in Table 2 was above 0.50 for all items. Based on the content of the questions in each factor, we called the three factors Patient-Centered Interaction, Outcome, and Care and Safety. The mean score of these three factors may be used as subscales. The results are shown in Table 2. In addition, the three dichotomous (yes/no) questions regarding received information about patient rights form a fourth subscale with a close to fair internal consistency (alpha of 0.64). We considered the structural validity and internal consistency as adequate.

**Table 2:** Factor structure*, internal consistency (Cronbach’s alpha) for factors, corrected item-total correlation, and descriptive imputed data for the final questionnaire (N = 96)

Factors and questions	Factor-loading	Corrected item-total correlation	Mean (SD)
**Factor 1: Patient-centered Interactions (alpha = 0.88, explains 26.2% of variance)**			3.03 (0.84)
12.Have you received information about possible medication side effects?	0.86	0.73	2.32 (1.19)
11.Have you received information about the effects of the medication?	0.83	0.72	2.84 (1.16)
1.Were you involved in planning your stay?	0.70	0.62	2.35 (1.36)
2.Have you been consulted about the treatment?	0.63	0.61	2.81 (1.24)
10.Have you received information about treatment options available to you?	0.63	0.61	2.95 (1.28)
9.Have you received information about how the therapists assessed your health condition?	0.60	0.58	3.10 (1.13)
15.Have you been involved in preparing your discharge?	0.59	0.61	3.17 (1.21)
13.Has the information been understandable?	0.56	0.58	3.38 (1.08)
4.Have nurses and other health personnel been able to help you?	0.51	0.63	3.81 (0.86)
**Factor 2: Outcome (alpha = 0.86, explains 19.2% of variance)**			3.34 (0.99)
5.Has your mental health improved?	0.83	0.85	3.22 (1.22)
16.Do you feel ready to be discharged now?	0.81	0.52	3.48 (1.21)
7.Have you become better able to master your daily tasks?	0.78	0.78	3.21 (1.20)
3.Has your doctor or psychologist been able to help you?	0.56	0.60	3.66 (1.03)
6.Has your physical health improved?	0.56	0.62	2.86 (1.62)
**Factor 3: Care and Safety (alpha = 0.76, explains 17.2% of variance)**			4.19 (0.68)
17.Would you return here if you needed a new stay?	0.79	0.63	4.06 (1.02)
8.Have you felt safe in the department?	0.79	0.64	4.16 (0.77)
14.Have the health personnel treated you with respect?	0.70	0.61	4.26 (0.59)

*) Factors 1–3 explain altogether 62.4% of the total variance in the 17 questions

### Construct validity

Table 3 shows the results from the construct validity test of the final questionnaire. For 19 of the 20 questions, more than half of the twenty-six participants sorted them to the correct dimension according to the factor structure. For 17 of these, more than two-thirds of the participants sorted them correctly. Question 4 (‘Have nurses and staff been able to help you?’) was sorted along with question 3 (‘Has your doctor or psychologist been able to help you?’) to the factor Outcome. These two questions have similar content, and the factor loadings of question 4 were almost as high on factor 2 as on factor 1. By taking both these findings into account, we concluded that question 4 belongs to the subscale “Outcome” together with question 3. With this correction, we considered the construct validity of the questionnaire to be adequate, and the internal consistency of the two subscales barely changed.

**Table 3:** Construct validity from item sorting (%) on factors by patients, clinicians and researchers (N = 26)

	Factors from the factor analysis			
Subscales and questions	Patient-centered Interaction	Outcome	Care and Safety	Information on Rights
**Subscale: Patient-centered Interaction**				
1.Were you involved in planning your stay?	**96.2**	0.0	3.8	0.0
2.Have you been consulted about the treatment?	**80.8**	0.0	3.8	15.4
9.Have you received information about how the therapists assessed your health condition?	**73.1**	7.7	0.0	19.2
10.Have you received information about treatment options available to you?	**50.0**	3.8	3.8	42.3
11.Have you received information about the effects of the medication?	**73.1**	3.8	7.7	15.4
12.Have you received information about possible medication side effects?	**73.1**	7.7	0.0	19.2
13.Has the information been understandable?	**69.2**	3.8	11.5	15.4
15.Have you been involved in preparing your discharge?	**92.3**	0.0	7.7	0.0
**Subscale: Outcome**				
3.Has your doctor or psychologist been able to help you?	7.7	**80.8**	11.5	0.0
4.Have nurses and other health personnel been able to help you?	7.7	**73.1**	19.2	0.0
5.Has your mental health improved?	0.0	**84.6**	15.4	0.0
6.Has your physical health improved?	3.8	**84.6**	11.5	0.0
7.Have you become better able to master your daily tasks?	11.5	**80.8**	3.8	3.8
16.Do you feel ready to be discharged now?	26.9	**57.7**	7.7	7.7
**Subscale: Care and Safety**				
8.Have you felt safe in the department?	11.5	3.8	**80.8**	3.8
14.Have the health personnel treated you with respect?	7.7	7.7	**80.8**	3.8
17.Would you return here if you needed a new stay?	7.7	23.1	**65.4**	3.8
**Subscale: Information on Rights**				
18.Have you received information about your right to access your medical record?	7.7	0.0	3.8	**88.5**
19.Have you been informed that you have opportunity to complain about the treatment?	7.7	0.0	3.8	**88.5**
20.Have you received information about your right to an individual plan?	15.4	0.0	7.7	**76.9**

### The final questionnaire

The final questionnaire, Patient Experiences in Psychiatric Departments for the Elderly (PEPDE), covers the following four subscales with adequate structural validity, internal consistency, and construct validity: Patient-centered Interaction (eight questions), Outcome (six questions), Care and Safety (three questions), and Information on Rights (three questions). The four subscales with questions are shown in Table 3. The questionnaire is brief, and the low number of missing responses indicates that it is feasible, well understood, and easy to complete by elderly patients. The final questionnaire has been translated into English with minor adjustments after back translation to Norwegian by a language translator firm. The English PEPDE with guidelines is available as an online supplementary material to this article.

## Discussion

### Contents of the questionnaire

We found that the final questionnaire PEPDE with 20 questions covered four dimensions with adequate measurement properties. These dimensions include most of the themes identified as relevant when we developed and tested the first version of the questionnaire and interviewed the patients. However, the final dimensions are fewer as identified themes clustered into fewer dimensions.

The final questionnaire contains questions from all themes defined in the first questionnaire, except questions about cooperation with family (next of kin). Based on comments reported from the patients in the first questionnaire, we removed these questions about information to family. Furthermore, we concluded that data on information to family or relatives should be collected by asking family or relatives directly.

The first subscale, Patient-centered Interaction, contains questions about patient involvement in planning the stay, treatment decisions, and preparations for discharge. Included are questions on whether the patient has received information on health assessment and available treatments options, as well as information on the effects of medication and possible side effects. This subscale measures patient experiences of modern patient-centered health care and shared decision-making.

Outcome, the second subscale, contains questions about improvements of physical and mental health, the improved ability to master daily tasks, and readiness for discharge. Two questions are about the help provided by doctors/psychologists and nurses or other health personnel. This subscale measures several aspects of improved health during the patient’s stay and the contributions from health professionals with different roles in the treatment.

The third subscale, Care and Safety, contains questions about being treated with respect, feeling safe in the department, and being inclined to return if in need of a new stay. This subscale measures the generally positive experience of being cared for and respected, reflecting the staff’s attitudes and ability to see and meet the patient as a person.

Information on Rights, the fourth subscale, includes whether the patient has received information on patients’ rights. This subscale measures aspects of health care that service user organizations have been much concerned with and where need for improvement is often measured [4, 12].

PEPDE has questions from six of the seven key domains identified in a review of PREMs in mental health care for adults (interpersonal relationships, respect and dignity, drug therapy, information, psychological care, and care environment) [4]. However, PEPDE has no question about care access and care coordination.

We have also compared the PEPDE to the Psychiatric Inpatient Patient Experience Questionnaire (PIPEQ) used in Norwegian national surveys in psychiatric inpatient departments for adults [14, 29]. The latest version of the PIPEQ has 17 questions and three subscales: Patient-centered Interactions (eight questions), Outcomes (five questions), and Structure and Facilities (four questions). The content of these subscales corresponds to a large extent with the PEPDE subscales, but there are some differences in the question contents. The five-point response scale in PIPEQ is identical to the response scale in PEPDE. However, the questions in PIPEQ are generally longer and perhaps less easy to understand. The Swedish Quality of Psychiatric Care In-Patient (QPC-IP) questionnaire has 30 more detailed questions with a four-step response scale, and six subscales with three to eight questions in each: Encounter, Participation, Discharge, Support, Seclude environment, and Secure environment [31]. The PEPDE has questions covering much of the same as the first three and the last of these subscales, but not the specific questions on Support and on Seclude environment. A few questions seem less relevant for elderly (e.g. ‘Help in finding occupation’).

### Methodological considerations

Our efforts to achieve an acceptable content validity were first to identify relevant domains and questions from the literature, the feedback from patients in the pilot study, and the discussions with clinicians and staff in the psychiatric inpatient department for the elderly in our hospital. Secondly, we collected data from Sample 1 on how the patients and the interviewers experienced each of the 37 questions in the first questionnaire. This collection was done systematically by adding an appendix to the questionnaire where the interviewer reported both comments from the patient and their own observations and reflections regarding each question. This detailed information was valuable to identifying questions that were less relevant or did not function well. These procedures, both in developing the first questionnaire and in examining the systematic feedback from patients and interviewers, helped us learn what was important to the patients, which questions to remove because they were unimportant or difficult to answer, and how the questions should be formulated.

The second and third steps of the revision process (removing questions to reduce the length of the questionnaire, improving the clarity of questions) were done without any new input from the patients. However, we followed certain guidelines to improve the questions by removing redundant words without changing the meaning of the question, especially for lengthy questions and questions that some patients had found difficult to understand. When deleting questions from the questionnaire, we looked for ones that were either partly or better covered by other questions. We believe that comparing the first questionnaire in Online Additional Table A and the final questionnaire in Table 1 shows that the final questionnaire is easier to understand and complete.

Testing the final questionnaire as described, we found that the questionnaire had adequate content validity, structural validity, internal consistency, and construct validity. This included the three measurement properties defined by COSMIN that have been tested most commonly for PREMs: internal consistency, structural validity and content validity [3]. With our sample size of 96 and a sample/question ratio 96:17 (5.6:1) the structural validity of the questionnaire may be considered as just about adequate according to the COSMIN criteria for patient-reported outcome measures [22]. However, it is desirable to have the factor structure confirmed with larger samples, as the necessary sample sizes for factor analyses vary due to various characteristics of the sample and of the statistical procedures [32–34]. As we did not find any PREM for psychiatric inpatient departments for the elderly, we could not test criterion validity by comparison to another established, similar instrument as recommended in the COSMIN standards. However, with the data from Sample 3, we did find adequate construct validity. Measurement properties that remain to be documented for PEPDE are measurement invariance, reliability by test-retest, measurement error, responsiveness, and cultural validity.

With 20 questions, the length of PEPDE is shorter than the median number of 27 questions in other PREMs found in a systematic review [3]. Nevertheless, PEPDE and its four subscales is close to the median number of five domains from that same review. As the inpatients in psychiatric departments for the elderly may be less capable than adults at completing longer questionnaires, it may be appropriate that PEPDE is shorter and easier to complete than the average PREM for adults in health services.

### Potential use of the questionnaire in clinical work and research

The Patients Experiences in Psychiatric Departments for the Elderly (PEPDE) questionnaire has been made available with a Creative Commons license. This license allows for free uncommercial use in its present form when citing this article, but it does not permit any commercial use. PEPDE and its guidelines for use are available as online supplementary material to this article.

The questionnaire may be used as feedback in inpatient departments for the elderly to further improve their quality of care. It may be used routinely or as an outcome for specific changes or interventions. Aggregating questionnaire data across inpatient departments may give annual statistics on patients’ experiences on a regional or national level.

The questionnaire may also be used in clinical and health service research studies to measure the patients’ experiences as an outcome of care or an aspect of the quality of care. Cross-sectional studies comparing patients’ experiences across sites may generate new knowledge about similarities and differences. Longitudinal studies with repeated data collection may test the changes over time in relation to changes in the provision of services. Clinical trials may study the effect of specific interventions on patients’ experiences.

It has been argued and experienced that interviews may function better than self-reported questionnaires for many elderly patients [7]. As described above, data collection with Sample 1 involved using the first questionnaire in an interview where the interviewer recorded the responses to the questionnaire while the patient read a copy of the questionnaire and response scale. We believe that PEPDE may be used both as a questionnaire completed by patients alone and as a structured interview for patients who may not be able to complete it alone. If using the interview method, one must consider whether the interviewer should be a person the patient does or does not know, also taking into account whether the patient feels free to give honest answers if the interviewer is among the staff providing treatment.

### Strengths and limitations

One strength from the development of this questionnaire was that a representative sample of patients and interviewers gave extensive feedback in the testing of the first questionnaire. This feedback provided a good base for the revision leading to the final questionnaire. There are several limitations, however. Content validity of the final questionnaire has yet to be evaluated by any external expert and we did not use any quantitative measure for content validity. It is desirable that structural validity is confirmed with a larger sample. Test-retest reliability has not been established, and questions on the involvement of family members (next of kin) are missing from the questionnaire. Testing for validity by comparison with similar established instruments was impossible due to the lack of any similar measurement tool for the elderly. Further analyses of additional measurement properties (including measurement invariance, measurement error, responsiveness) remain, and the final questionnaire should be tested in a broader context.

## Conclusion

The questionnaire Patient Experiences in Psychiatric Departments for Elderly (PEPDE) has been developed addressing the situation, experiences, and concerns of such patients. The questionnaire is brief and easy to complete, and it has adequate structural validity and cover four core dimensions with adequate internal consistency. Low number of missing responses indicates that it is well understood and feasible. It is made available with instructions and under a Creative Commons license for use for feedback, quality improvement and research in psychiatric departments for elderly. As far as we know, this is the first PREM for inpatients in psychiatric departments for elderly, and it may hopefully fill the gap for such a questionnaire and contribute to better knowledge of the experiences of these patients and how their stays and treatments may be improved.

## Electronic supplementary material

Below is the link to the electronic supplementary material.

Supplementary Material 1 Online Additional Table A

Supplementary Material 2 The questionnaire PEPDE with brief guidelines

## Data Availability

The datasets used and/or analyzed during the current study are available from the corresponding author on reasonable request.

## Abbreviations

COSMIN: Consensus-based Standards for the selection of health Measurement Instruments
PIPEQ: Psychiatric Inpatient Patient Experience Questionnaire
PEPDE: Patient Experiences in Psychiatric Departments for Elderly
PREM: Patient-reported experience measures
QPC-IP: Quality of Psychiatric Care In-Patient questionnaire
