# Supplementary material for: Patient experiences in psychiatric departments for the elderly (PEPDE): development, properties, and use of a brief questionnaire
Source: BMC Psychiatry. 2023 Mar 16;23:173. doi: 10.1186/s12888-023-04633-y (PMC10021934; doi:10.1186/s12888-023-04633-y)
Supplement: Supplementary file 1 — Supplementary Material 1 Online Additional Table A [file 12888_2023_4633_MOESM1_ESM.docx]

**Supplementary Table A. Decisions on each questions in the first questionnaire during the revision to form the final questionnaire, and the numbering of the retained questions in the final questionnaire**

| **Themes and questions** | **Decision** | **No. in final questionnaire** |
| --- | --- | --- |
| **A: Questions about general experiences of being here** |  |  |
| 1. Are you satisfied with the services you have received in the department? | 2. Removed to reduce items |  |
| 2. Were you involved in planning your stay? | 4. Retained unchanged | 1 |
| 3. Have you felt safe in the department? | 4. Retained unchanged | 8 |
| 4. Did you feel lonely in the ward? | 1. Removed due to feedback |  |
| 5. Did you get to decide how much you wanted to be with the other patients? | 2. Removed to reduce items |  |
| 6. Did you have anything meaningful to do? | 1. Removed due to feedback |  |
| 7. Have you felt discomfort by having limited freedom of movement or other restrictions during your stay? | 1. Removed due to feedback |  |
| 8. Were you asked for advice regarding the treatment (milieu therapy, conversations, medication, group activities)? | 3. Retained, improved clarity | 2 |
| **B: Questions about your benefit from being here** |  |  |
| 9. Did you get the treatment you needed? | 1. Removed due to feedback |  |
| 10. Has your mental health improved? | 4. Retained unchanged | 5 |
| 11. Has your physical health improved? | 4. Retained unchanged | 6 |
| 12. Have you become better able to master your daily tasks? | 4. Retained unchanged | 7 |
| 13. Has it become easier for you to be with others? | 1. Removed due to feedback |  |
| 14. Have you received any treatment that has not been good for you (eg ECT, medication, group therapy, ward milieu)? | 2. Removed to reduce items |  |
| **C: Questions about how you have experienced the staff** |  |  |
| 15. Have staff ever treated you condescendingly or disrespectfully? | 3. Retained, improved clarity | 14 |
| 16. Were nurses or other staff available when you needed them? (applies to staff who are not doctors or psychologists) | 2. Removed to reduce items |  |
| 17. Do you think that nurses and other staff could help you with what you came here for? (not doctors or psychologists) | 3. Retained, improved clarity | 4 |
| 18. Did your doctor or psychologist have enough time for you? | 1. Removed due to feedback |  |
| 19. Did you feel that your doctor or psychologist could help you with what you came here for? | 3. Retained, improved clarity | 3 |
| **D: Questions about information** |  |  |
| 20. Have you received information about how the therapists assessed your health condition? | 4. Retained unchanged | 9 |
| 21. Have you received information about the treatment options available to you? | 4. Retained unchanged | 10 |
| 22. Has the information been understandable? | 4. Retained unchanged | 13 |
| 23. Have you received information about the effects of the medication? | 4. Retained unchanged | 11 |
| 24. Have you received information about possible medication side effects? | 4. Retained unchanged | 12 |
| 25. Did you get information about how to take the medication? | 2. Removed to reduce items |  |
| 26. Were you asked for advice about the medication? | 2. Removed to reduce items |  |
| **E: Information on patients’ rights** |  |  |
| 27. Have you received information about the opportunities you have to complain about the treatment? (county doctor, control commission, patient ombudsman?) | 3. Retained, improved clarity | 19 |
| 28. Have you received information about your right to access your medical record? | 4. Retained unchanged | 18 |
| 29. Have you received information about your right to an individual plan | 4. Retained unchanged | 20 |
| **F: Questions about cooperation with family/relatives** |  |  |
| 30. Did you want your next of kin to be informed about your illness? | 1. Removed due to feedback |  |
| 31. Did your next of kin receive information about your illness? | 1. Removed due to feedback |  |
| 32. Did your next of kin receive information about where you can get the help you need after discharge? | 1. Removed due to feedback |  |
| **G: Questions about the discharge and benefits of the stay in the department** |  |  |
| 33. Have you been involved in preparing your discharge? | 4. Retained unchanged | 15 |
| 34. Is the plan after discharge well enough prepared? | 2. Removed to reduce items |  |
| 35. Do you feel ready to be discharged now? | 4. Retained unchanged | 16 |
| 36. Do you experience that the admission was necessary? | 2. Removed to reduce items |  |
| 37. If you should need a new stay in a psychiatric inpatient department later, how would you like to come back here? | 3. Retained, improved clarity | 17 |
